# An Archaeal Chitinase With a Secondary Capacity for Catalyzing Cellulose and Its Biotechnological Applications in Shell and Straw Degradation

**DOI:** 10.3389/fmicb.2019.01253

**Published:** 2019-06-11

**Authors:** Lina Chen, Yi Wei, Mao Shi, Zhengqun Li, Shi-Hong Zhang

**Affiliations:** ^1^ College of Plant Sciences, Jilin University, Changchun, China; ^2^ College of Food Science and Engineering, Changchun University, Changchun, China; ^3^ Jilin Provincial Center for Disease Control and Prevention, Changchun, China

**Keywords:** thermostable enzyme, chitinase, cellulose catalysis, yeast expression system, shell and straw degradation, response surface methodology

## Abstract

Numerous thermostable enzymes have been reported from the hyperthermophilic archaeon *Thermococcus kodakarensis* KOD1, which made it an attractive resource for gene cloning. This research reported a glycosyl hydrolase (Tk-ChiA) form *T. Kodakarensis* with dual hydrolytic activity due to the presence of three binding domains with affinity toward chitin and cellulose. The *Tk-ChiA* gene was cloned and expressed on *Pichia pastoris* GS115. The molecular weight of the purified Tk-ChiA is about 130.0 kDa. By using chitosan, CMC-Na and other polysaccharides as substrates, we confirmed that Tk-ChiA with dual hydrolysis activity preferably hydrolyzes both chitosan and CMC-Na. Purified Tk-ChiA showed maximal activity for hydrolyzing CMC-Na at temperature 65°C and pH 7.0. It showed thermal stability on incubation for 4 h at temperatures ranging from 70 to 80°C and remained more than 40% of its maximum activity after pre-incubation at 100°C for 4 h. Particularly, Tk-ChiA is capable of degrading shrimp shell and rice straw through scanning electron microscopy (SEM) and Fourier transform infrared spectroscopy (FT-IR) analysis. The main factors affecting shell and straw degradation were determined to be reaction time and temperature; and both factors were optimized by central composite design (CCD) of response surface methodology (RSM) to enhance the efficiency of degradation. Our findings suggest that Tk-ChiA with dual thermostable hydrolytic activities maybe a promising hydrolase for shell and straw waste treatment, conversion, and utilization.

## Introduction

Chitin is an insoluble linear polymer of β-1,4-linked N-acetylglucosamine (GlcNAc) that is different from cellulose, which consists of non-branched β-1,4-linked glucose. Chitin is frequently present in the cell walls of fungi, the cuticles of insects (Hanazono et al., 2016), and the shells of shrimp. However, cellulose is the major polysaccharide of plant cell walls and is thus the most abundant natural polymer. Cellulose is present in various types of straw and is a valuable natural resource (Li et al., 2018). Approximately, 10^14^–10^15^ tons of cellulose is produced annually in the form of raw materials derived from straw, wood, cotton, reeds, hemp, and mulberry (Bayer et al., 1998; Zarafeta et al., 2016). In contrast, the annual amount of chitin production is estimated to be approximately 10^10^–10^11^ tons (Gooday, 1990), which makes it the second most abundant natural polymer. Chitin and cellulose degradation are excellent potential sources of biofuel, so industrial utilization of chitin and cellulose has become a popular area of research aimed at solving the problems of increasing energy costs, pollution, and global warming (Wan et al., 2011; Fan et al., 2013). Enzymatic degradation of chitin and cellulose is preferred over other methods because of its lack of environmental impact (Zhang et al., 2012).

Chitinase [EC3.2.1.14] enzymes have the capacity to hydrolyze the β-1,4-linkage between GlcNAc molecules in chitin and are expressed by bacteria, fungi, animals, plants (Adrangi and Faramarzi, 2013; Hamid et al., 2013), and archaea (Huber et al., 1995; Tanaka et al., 1999). Chitinases are classified into glycoside hydrolase family 18 (GH18) and 19 (GH19) in the carbohydrate-active enzymes (CAZy) database^1^ based on the amino acid sequences of their catalytic modules (Henrissat, 1991; Levasseur et al., 2013). GH18 chitinases always contain one catalytic domain and several auxiliary domains, including chitin-binding domains and an N-terminal signal peptide region (Hartl et al., 2012). Among the domains of GH18 chitinases, the catalytic domain is most important for chitin hydrolysis and contains the following highly conserved motif: DXXDXDXE (Henrissat and Bairoch, 1996). The chitin-binding domains of GH18 chitinases belong to carbohydrate binding module (CBM) families 5, 12, 14, 18, and 50.

The ability of chitinases to catalyze chitin degradation allows the utilization of common sources of chitin, such as shrimp shell, as energy sources in industrial applications. For the purpose of optimization of industrial processes, it is necessary to search for chitinases with very high activity levels under high temperature conditions, which promote high catalytic rates (Niehaus et al., 1999; Liang et al., 2011; Frock and Kelly, 2012; Keller et al., 2015). *Thermococcus kodakarensis* KOD1 is a hyperthermophilic archaeon with a fully sequenced genome (Fukui et al., 2005). Over the last decade, various thermophilic enzymes from *T. kodakarensis* KOD1 have been reported, including a lysophospholipase (Cui et al., 2012), an amylopullulanase (Guan et al., 2013), a cyclodextrinase (Sun et al., 2015), and proteases (Foophow et al., 2010; Jia et al., 2015). As a hyperthermophilic anaerobe, *T. kodakarensis* KOD1 is a potential thermophilic enzyme source for industrial applications.

*T. kodakarensis* chitinase Tk-ChiA is encoded by the *Tk1765* gene (Tanaka et al., 1999, 2001). Tk-ChiA is a thermostable enzyme with an optimal temperature of 80°C, which suggests that it could be useful for chitin degradation in high-temperature industrial processes. Tk-ChiA consists of two GH18 catalytic domains, as well as N-terminal and C-terminal domains that show exo-chitinase and endo-chitinase functionality, respectively. In addition, Tk-ChiA has three substrate binding domains, CBD1, CBD2, and CBD3, which are considered to bind chitin. However, these three domains show sequence similarity with the cellulose binding domains of various cellulases. CBD1 is a family V type carbohydrate binding domain that is capable of cellulose binding (Tomme et al., 1998). CBD2 and CBD3 are classed as family II type carbohydrate binding domains. Binding domains play an important role in determining the catalysis ability of enzymes (Jendrossek and Handrick, 2002). Strikingly, CBD2 and CBD3 can bind to chitin and cellulose (Hanazono et al., 2016). However, CBD2 and CBD3 are unable to bind to xylan and amylopectin. These cellulose binding domains of Tk-ChiA allow it to perform the secondary function of catalyzing cellulose degradation. Cellulases hydrolyze the β-1,4 linkages in cellulose and are the main catalysts involved in enzymatic hydrolysis. Cellulases function in multi-enzyme systems comprising an endoglucanase (EC3.2.1.4), a cellobiohydrolase (avicelase, EC3.2.1.91), and a β-d-glucosidase (EC3.2.1.21), which synergistically hydrolyze cellulose into monomeric glucose units (Chang et al., 2016). Cellulases are potentially useful in the biofuel industry because cellulose is the most abundant energy source on Earth (Zhang and Zhang, 2013). Therefore, a chitinase with the dual capacities for hydrolyzing chitin and cellulose could be useful for biofuel industrial applications, but such an enzyme has not been reported.

In this study, we purified Tk-ChiA expressed in the *Pichia pastoris* system to investigate its secondary ability for cellulose degradation with a focus on industrial applications.

## Materials and Methods

### Strains, Vectors, and Cultural Media

*T. kodakarensis* KOD1, which was kindly donated by the Japan Collection of Microorganisms, RIKEN BioResource Center, Japan, was used to isolate genomic DNA. *T. kodakarensis* KOD1 was cultured in 280 *Thermococcus* medium (Fukui et al., 2005). *P. pastoris* GS115 and pPIC9K plasmid were used as the host and expression vector, respectively. The vector pPIC9K^2^, contains α-factor signal peptide sequence and a strong promoter controlling recombinant gene expression, the methanol-inducible alcohol oxidase gene (*AOX1*).

### Cloning, Expression, and Purification of Tk-ChiA From *T. kodakarensis* KOD1

The gene encoding Tk-ChiA (*Tk1765*) was amplified *via* PCR using DNA as the template (forward primer, 5′-CCG GAA TTC CAT CAT CAC CAT CAC CAT CTG AAG CTT ACG TAGA ATT CGA GAG CGT AAG CCT G-3′; reverse primer, 5′-TTG CGG CCG CGC GAA TTA ATT CGC GGC CGC TCA AAC TGG AAC TGC AA CTG-3′). The endonuclease sites *EcoRI* and *NotI* were introduced as underlined. The PCR product of *Tk1765* was purified with a DNA purification kit (TianGen, Beijing, China). The purified PCR product of *Tk1765* and vector pPIC9K was digested with *EcoRI* and *NotI,* after which they were ligated using T4 ligase (Takara, Dalian, China). The correct recombination plasmid pPIC9K-*AgCMAase* was linearized using *Bgl*II and transformed into *P. pastoris* GS115 competent cells by electroporation.

His^+^ transformants were recovered on MD plates at 30°C for 3 or 4 days (until single colonies appeared) and placed into shaking tubes for enzyme production according to the protocol described in the *Pichia* manual^3^. The recombinant *P. pastoris* GS115 containing pPIC9K-*Tk1765* was grown at 30°C in 250 ml BMGY medium in a 1-L shaking flask until the cell density reached an OD_600_ of 4.0. Cells were collected, re-suspended in 50 ml BMMY medium with 0.5% (v/v) methanol, and cultured at 25°C for 3 days with shaking (250 rpm).

The fermentation broth was centrifuged at 5,000 × *g* for 10 min and the supernatant was collected. First, the supernatant was concentrated ∼5-folds using lysis buffer containing 50 mM Tris-HCl (pH 8.0), 300 mM NaCl, 20 mM 2-mercaptoethanol by ultrafiltration in a Vivaflow 20 ultrafiltration membrane (50 kDa cut off; Sartorius Stedim Biotech, Germany). The concentrated supernantant was loaded onto a Ni^2+^-NTA column. After washing the column with lysis buffer, a linear gradient of imidazole (5–500 mM) in the lysis buffer was used to elute the proteins. Fractions having chitinase and cellulose activity were pooled and desalted using 0.2 M sodium acetate buffer (pH 5.0) *via* dialysis.

### SDS-PAGE and Zymogram Analysis

The purified AgCMCase was visualized after separation by 7% sodium dodecyl sulfate polyacrylamide gel electrophoresis (SDS-PAGE). The protein concentration was measured using a Protein Assay Kit (Bio-Rad, Hercules, CA, USA).

According to previously described methods (Li et al., 2012; Li and Yu, 2013a), zymogram analysis was performed by using a 0.1% CMC-Na (w/v) incorporated into the 7% polyacrylamide gel. The CMC-Na was pre-mixed with polyacrylamide during gel preparation. After electrophoresis, the gel was washed 3 times for 30 min in renaturation buffer (50 mM sodium phosphate buffer, pH 7.0). Then it was left in reaction buffer containing 50 mM Tris-HCl (pH 7.0) at 65°C for 1 h. The enzyme band was visualized by staining with 1% (w/v) Congo red (Lee et al., 2014).

### Enzymatic Activity Assay

The purified enzyme was used to characterize the properties of Tk-ChiA. The activity of the enzyme was measured using chitosan, sodium carboxymethyl cellulose (CMC-Na), pullulan, starch α-glucan from barley and Avicel as substrates. The enzymatic activity of Tk-ChiA was assessed by the 3,5-dinitrosalicylic acid (DNS, DingGuo, Beijing, China) method (Miller, 1959) to measure the amount of reducing sugars released from substrates. The reaction was conducted in a mixture of appropriately diluted enzyme solution and 0.6% (w/v) substrates dissolved in 50 mM citrate-phosphate buffer (pH 8.0) in a final volume of 0.25 ml, which was incubated at the optimum reaction temperature for 1 h. The reaction was stopped by the addition of 0.25 ml DNS. The treated sample was boiled for 10 min, the color was allowed to stabilize, and the absorbance was measured at 540 nm. The enzymatic activity was calculated from a calibration curve constructed using glucose as a standard. One unit of enzyme activity was defined as the amount of enzyme required to release 1 mM of reducing sugars from the substrate per minute.

The effect of pH on the CMC-Na hydrolysis activity of purified Tk-ChiA was determined using the DNS method described above. The reaction was carried out for 30 min at 65°C in buffers with various pH levels (4.0–13.0). The buffers used in this experiment are shown in Supplementary Table S1. The optimal temperature for CMC-Na degradation by Tk-ChiA was determined by testing temperatures ranging from 30 to 90°C for 30 min. All assays were performed at the optimal pH. Thermostability was determined by measuring the residual enzyme activity level after pre-incubation at 70, 80, 90, and 100°C for varying periods of time without a substrate. After various time intervals, samples were withdrawn, and the enzymatic activity of the sample was measured under optimal reaction conditions.

### Scanning Electron Microscopy (SEM)

SEM was conducted at magnification 15,009 for both native and enzymatic treatment samples (shrimp shell and rice straw) using a JEOLJSM-7500 scanning electron microscope.

### Fourier Transform Infrared Spectroscopy (FT-IR)

The samples of shrimp shell and rice straw were freeze-dried, smashed into a powder, and mixed with potassium bromide (1:200). The infrared spectrum of the prepared samples was recorded by FT-IR (Thermo Nicolet NEXUS 670, USA) between 4,500 and 500 cm^−1^.

### Statistical Experimental Designs

Reaction time (*X*_1_), temperature (*X*_2_), pH (*X*_3_), and enzyme dosage (*X*_4_) were selected as important factors influencing enzyme activity, and their effects on reducing sugar production were evaluated using an experiment with a Box-Behnken factorial design (BBD). All factors were tested at three levels (Table 1). The results are described using the following equation:

**Table 1:** Experimental design.

	Levels		
Factors	−1	0	1
*X*_1_ *–* time (h)	3	4	5
*X*_2_ *–* temperature (°C)	60	70	80
*X*_3_ *–* pH	7	8	9
*X*_4_ *–* enzyme dosage (ml)	1.5	2	2.5

(1) $$\begin{array}{l} Y=\beta_{0}+\beta_{1}X_{1}+\beta_{2}X_{2}+\beta_{3}X_{3}+\beta_{4}X_{4}+\beta_{11}X_{1}^{2}+\beta_{22}X_{2}^{2}\\ \;+\beta_{33}X_{3}^{2}+\beta_{44}X_{4}^{2}+\beta_{12}X_{1}X_{2}+\beta_{13}X_{1}X_{3}+\beta_{14}X_{1}X_{4}\\ \;+\beta_{23}X_{2}X_{3}+\beta_{24}X_{2}X_{4}+\beta_{34}X_{3}X_{4} \end{array}$$

where *Y* is the dependent variable (reducing sugar production); *β*_0_ is the model constant; *β*_1_, *β*_2_, *β*_3_, and *β*_4_ are linear coefficients; *β*_11_, *β*_22_, *β*_33_, and *β*_44_ are squared coefficients; *β*_12_, *β*_13_, *β*_14_, *β*_23_, *β*_24_, and *β*_34_ are interaction coefficients.

### Central Composite Design (CCD)

CCD of response surface methodology (RSM) was used to optimize the two factors that most significantly influenced reducing sugar production. Each factor in the design matrix was tested at five different levels (−1.414, −1, 0, 1, and 1.414). Thirteen runs of two variables for enhancing reducing sugar production (*Y*) were conducted in this experimental design. The effects of the selected variables on the response were calculated by the following second-order model:

(2) $$Y=\beta_{0}+\beta_{1}X_{1}+\beta_{2}X_{2}+\beta_{11}X_{1}^{2}+\beta_{22}X_{2}^{2}+\beta_{12}X_{1}X_{2}$$

where *Y* is the dependent variable (reducing sugar production); *β*_0_ is the model constant; *β*_1_ and *β*_2_ are linear coefficients; *β*_11_ and *β*_22_ are squared coefficients; *β*_12_ is an interaction coefficient.

### Statistical Analysis

Statistical optimization was performed using Design Expert (version 7.0, STATEASE Inc., Minneapolis, MN, USA). Data analyses were carried out by analysis of variance (ANOVA) using Microsoft EXCEL and GraphPad Prism 5. ANOVA was used to determine statistically significant differences. All experiments were performed in triplicate.

## Results

### Expression and Purification of Tk-ChiA in the *P. pastoris* System

**Figure 1:**
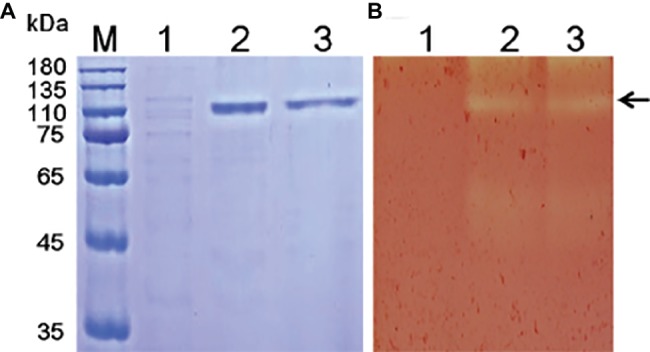
SDS-PAGE **(A)** and zymogram **(B)** analysis of Tk-ChiA from *T. kodakarensis* KOD1. Lane M, protein marker (GenStar, Beijing); Line 1, crude extract of *P. pastoris* GS115 transformed empty plasmid (pPIC9K); Lane 2, crude protein extract from the supernatant of the recombinant strain transformed pPIC9K*-Tk1765* plasmid; Lane 3, purified recombinant Tk-ChiA. The arrow indicates the position of Tk-ChiA.

Various methods have been tested for the identification of functional enzyme genes, but expression assays in *Escherichia coli* were found to be inefficient due to the formation of intracellular proteins or inclusion bodies (Bayer et al., 1998; Chang et al., 2016). *P*. *pastoris* is frequently used to express various heterogeneous proteins, including chitinases, and has many of the advantages of other expression systems. In addition, *P*. *pastoris* is faster, easier, and less expensive than many other expression systems. To study the function of Tk-ChiA, a gene fragment (*Tk1765*) encoding the enzyme without the signal peptide was expressed in the *P*. *pastoris* system (Figure 1). We selected the positive recombinant strain *P*. *pastoris* with the highest Tk-ChiA activity that was fermented in a 1 L flask at 30°C for 72 h with 0.5% (v/v) methanol added every 24 h. The yield of crude enzyme product and enzyme activity reached 0.31 mg/ml and 50.49 U/ml after induction for 72 h, respectively (Supplementary Table S6). As indicated in lane 1 of the SDS-PAGE gel, there was only one major protein band at approximately 130 kD in the fermentation broth sample, suggesting that the recombinant Tk-ChiA secreted by *P*. *pastoris* did not need further purification. The mass of the protein band matched the predicted molecular mass (134.27 kDa) of Tk-ChiA. The crude Tk-ChiA enzyme was further purified from the cell-free medium with a single-step process using a Ni^2+^-NTA column, which produced a single band in lane 2 of the SDS-PAGE gel (Figure 1).

### Secondary Cellulose Hydrolytic Activity of Tk-ChiA

**Figure 2:**
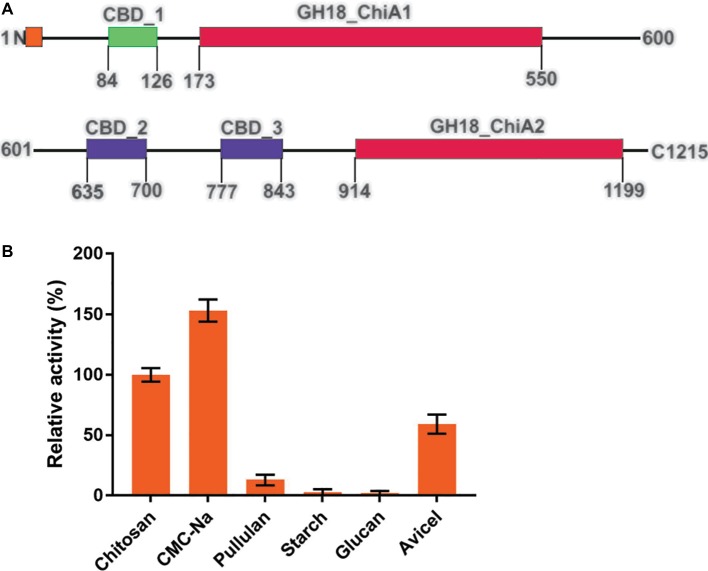
Structural features of Tk-ChiA and the hydrolytic activity of the enzyme toward various substrates. **(A)** Domain architecture of Tk-ChiA. A putative signal sequence and the enzyme catalytic domains (GH18_ChiA1, 2) are shown in orange and red, respectively. The chitin-binding domain (CBD1) and chitin/cellulose-binding domains (CBD2, 3) are shown in green and purple, respectively. **(B)** Hydrolytic activity of Tk-ChiA for chitosan, CMC-Na, pullulan, starch, glucan and Avicel. The experiment was conducted in triplicate. Assays were performed as described in the section “Materials and Methods.”

Tk-ChiA possesses dual GH18 catalytic domains and triple CBDs (Figure 2A; Supplementary Figure S1; Tanaka et al., 1999). Based on the sequence aliment of CBD2 and CBD3, they are classed as family II cellulose binding domains, which include four strictly conserved tryptophan residues (Din et al., 1994) and are similar to the *Butyrivibrio fibrisolvens* H17c endoglucanase cellulose binding domain (Berger et al., 1989). CBD2 and CBD3 were also expressed and purified independently to investigate their binding capacity with several substrates (Hanazono et al., 2016). CBD2 and CBD3 were found to bind to cellulose, suggesting that Tk-ChiA possesses a secondary cellulose hydrolysis function. Zymographic analysis showed a significant activity band that corresponded to Tk-ChiA (about 130 kDa) (Figure 1B).

To test the substrate selectivity of Tk-ChiA, various polysaccharide substrates subjected to degradation, including chitosan, Sodium carboxymethyl cellulose (CMC-Na), pullulan, soluble starch, glucan, and Avicel. Corresponding to previous studies, Tk-ChiA preferably hydrolyzed chitosan, and the chitosan catalysis activity of Tk-ChiA was scaled to 100%. Strikingly, Tk-ChiA also had a high level of CMC-Na hydrolysis activity (approximately 152% compared to chitosan) (Figure 2B). Tk-ChiA showed nearly no effect on pullulan, soluble starch, and glucan. These results show that Tk-ChiA preferably hydrolyzes the β-1,4-linkage in chitin and cellulose. However, this enzyme has low catalysis activity for β-1,3-1,4- linkages, such as that found in glucan. In addition, Tk-ChiA degraded Avicel, a type of crystalline cellulose (approximately 60% activity compared to chitosan), indicating that it has dual exo-1,4-β-glucanases and endo-1,4-β-glucanases functions (Park et al., 2012; Li et al., 2018).

### Effect of Temperature and pH on Cellulose Hydrolysis by Tk-ChiA

**Figure 3:**
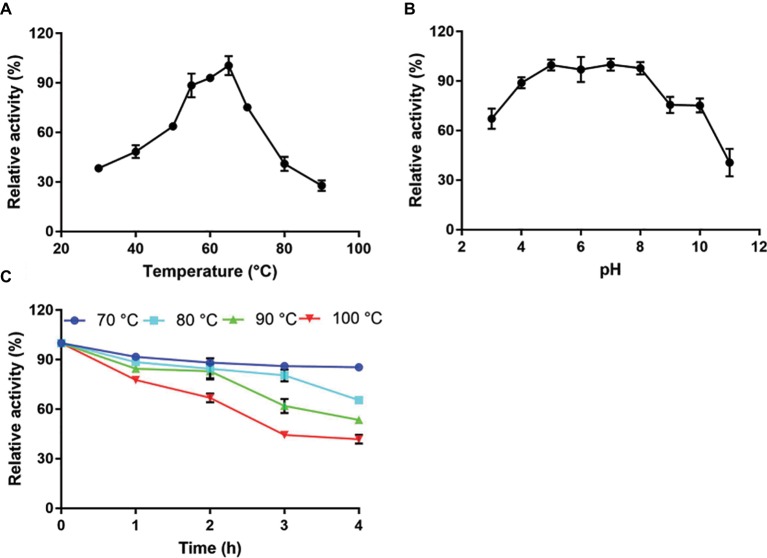
Characterization of CMC-Na hydrolysis by purified Tk-ChiA. **(A)** Influence of temperature on Tk-ChiA activity. Enzyme activity levels at various temperatures were assayed at pH 7.0 for 1 h using CMC-Na as the substrate. Values obtained at 65°C were set as 100%. **(B)** Effect of pH on Tk-ChiA activity. Enzyme activity levels at various pH values were assayed at 65°C for 1 h using CMC-Na as the substrate. Values obtained at pH 7.0 were set as 100%. **(C)** Thermostability of Tk-ChiA. Residual activity was assayed using CMC-Na as the substrates after pre-incubation without a substrate at 70°C (circle), 80°C (square), 90°C (triangle), and 100°C (inverted triangle) for different periods of time.

Recombinant full-length Tk-ChiA exhibited maximal cellulose hydrolysis activity at 65°C when CMC-Na was used as the substrate (Figure 3A). The activity of purified Tk-ChiA was detected from 30 to 90°C, and it remained high (>50% of peak activity) at temperatures ranging from 40 to 70°C (Figure 3A). Tk-ChiA showed approximately 40% of maximum cellulose hydrolysis activity at 80°C, which was also found to be the optimal temperature for chitin degradation (Tanaka et al., 1999). Therefore, Tk-ChiA can efficiently hydrolyze chitin and cellulose simultaneously.

To study the effect of pH on the cellulose degradation activity of purified Tk-ChiA, the enzyme and CMC-Na were combined at pH values between 3.0 and 11.0 at the optimal temperature of 65°C. The purified enzyme showed maximal activity for hydrolyzing CMC-Na at pH 7.0 and maintained more than 90% of its maximum activity at pH 5.0–8.0 (Figure 3B). Tk-ChiA showed low activity (40% of peak activity) at pH 11.0. The remarkable activity level of Tk-ChiA at a relatively wide range of pH values shows that it is suitable for cellulose degradation applications in alkaline and acidic environments.

The thermal stability of Tk-ChiA was tested by assessing its activity level under optimal reaction conditions (65°C, pH 7.0) after pre-incubation of the enzyme at 70–100°C for 4 h. The CMC-Na hydrolysis activity of Tk-ChiA remained high following exposure to high temperatures (Figure 3C). After pre-incubation at 70°C for 4 h, Tk-ChiA showed nearly full activity. After pre-incubation at 80°C for 4 h, Tk-ChiA showed a 30% loss of activity. After pre-incubation at 100°C for 4 h, Tk-ChiA retained more than 40% of its original activity. These results indicate that Tk-ChiA is a thermostable enzyme that is capable of hydrolyzing cellulose and thus has potential industrial applications under high temperature conditions.

### The Ability of Tk-ChiA to Degrade Shrimp Shell and Rice Straw

**Figure 4:**
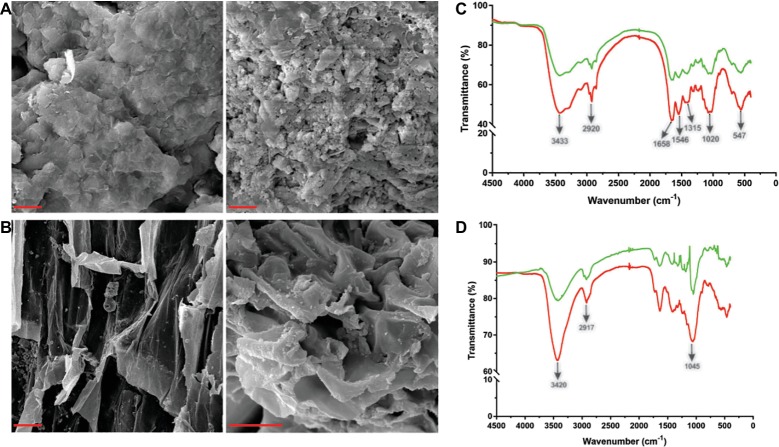
Scanning electron microscopy (SEM) and Fourier transform infrared spectroscopy (FT-IR) analysis of shrimp shell and rice straw incubated with Tk-ChiA. **(A)** After incubation of Tk-ChiA and shrimp shell at the optimal enzyme reaction conditions (pH 7.0, 65°C) for 5 h, the sample was observed by SEM with 1,000× amplification (right). The control sample was exposed to an equal volume of buffer (left). Scale bar (red), 5 μm. **(B)** After incubation of Tk-ChiA and rice straw at the optimal enzyme reaction conditions (pH 7.0, 65°C) for 5 h, the sample was observed by SEM with 1,000× amplification (right). The control sample was exposed to an equal volume of buffer (left). Scale bar (red), 5 μm. FT-IR spectra of shrimp shell **(C)** and rice straw **(D)** with enzymatic treatment (red) and without enzymatic treatment (green).

Tk-ChiA is a thermostable enzyme that can hydrolyze both chitin (Tanaka et al., 1999) and cellulose (Figure 2B). Therefore, Tk-ChiA could be suitable for industrial applications such as shrimp shell and straw degradation. Therefore, we incubated Tk-ChiA with shrimp shell and rice straw under optimal reaction conditions and assessed its effects. SEM indicated that shrimp shell and rice straw incubated with Tk-ChiA were obviously fractured (Figures 4A,B right) compared to shrimp shell and rice straw incubated without the enzyme (Figures 4A,B left). The shrimp shell incubated without Tk-ChiA had a smooth and rigid microfibrillar crystalline surface and an intact structure. In contrast, shrimp shell incubated with Tk-ChiA showed perforations and anomalous morphology (Figure 4A). The surface of rice straw that was not exposed to Tk-ChiA was smooth, uniform, and highly ordered. However, the structure of rice straw treated with Tk-ChiA was uneven, unsmooth, rough, and rugged (Figure 4B). These results demonstrate that both shrimp shell and rice straw can be degraded by Tk-ChiA.

The ability of Tk-ChiA to hydrolyze shrimp shell and rice straw was confirmed by FT-IR analysis. FT-IR spectroscopy was used to assess changes in the functional groups and structure of shrimp shell chitin and rice straw cellulose after Tk-ChiA treatment compared to untreated substrates. The FT-IR spectra of chitin were characterized by three significant amide bands at 1,658, 1,546, and 1,315 cm^−1^, which correspond to amide Ι (C═O stretching), amide ΙΙ (─NH2 stretching) and amide ΙΙΙ (C─N stretching), respectively (Liu et al., 2012). The band at 3433 cm^−1^ corresponded to O─H stretching. The band located at 1020 cm^−1^ between 890 and 1,156 cm^−1^ corresponded to C─O─C stretching of the β-glyosidic bond in chitin (Povea et al., 2011). These characteristic absorption peaks diminished after enzymatic treatment (Figure 4C), indicating that chitin was degraded by Tk-ChiA.

The peak of CH_2_ stretching located near 2,900 cm^−1^ and the peak of ─OH stretching located near 3,400 cm^−1^ are distinguishing features of cellulose (Sun et al., 2007) that indicate its most important functional groups. The peak at 3420 cm^−1^ that corresponded to the stretching of ─OH groups was attenuated after Tk-ChiA treatment (Figure 4D), suggesting that the partial hydrogen bond of cellulose was destroyed. The peak located at 2917 cm^−1^ that corresponded to the CH_2_ stretching decreased in intensity, indicating that the methyl and methylene groups of cellulose were broken (He et al., 2008). The peaks located at 900–1,100 cm^−1^ corresponded to C─O─C stretching at the β-1,4-glycosidic linkages (Hinterstoisser et al., 2001; Cao and Tan, 2004; He et al., 2008). The peak located at 1405 cm^−1^ was diminished after incubation with Tk-ChiA (Figure 4D), suggesting that the β-1,4-glycosidic linkages in rice straw cellulose were hydrolyzed.

### Reaction Time and Temperature Are the Main Factors Affecting Reducing Sugar Production

Straw and shell wastes are abundant sources of substrates that are suitable for degradation by Tk-ChiA to release reducing sugar. Statistical tests (Mabilia et al., 2010; Zhang and Jia, 2018) were used to study the effects of different reaction parameters on reducing sugar production. First, three levels of reaction time (*X*_1_), temperature (*X*_2_), pH (*X*_3_), and enzyme dosage (*X*_4_) were selected (Table 1). To evaluate the effects of these four factors and identify the main factors influencing the release of reducing sugar rice straw degradation, a Box–Behnken factorial design experiment was performed. Twenty-nine runs were conducted to analyze the effects of the four variables on rice straw degradation to release reducing sugar. The results of this analysis are shown in Table 2. A model was obtained by regression analysis as follows:

**Table 2:** Results of the fractional factorial design matrix of rice straw degradation.

Running number	*X*_1_	*X*_2_	*X*_3_	*X*_4_	Reducing sugar yield (%)
1	0	0	1	−1	1.71
2	1	−1	0	0	1.28
3	0	0	0	0	1.43
4	−1	−1	0	0	1.23
5	0	0	0	0	1.7
6	0	0	−1	1	1.78
7	0	1	0	−1	1.66
8	1	0	−1	0	1.53
9	0	−1	−1	0	1.62
10	0	−1	1	0	1.33
11	−1	0	−1	0	0.83
12	−1	0	1	0	1.08
13	0	0	0	0	1.74
14	0	0	0	0	1.69
15	0	1	0	1	1.79
16	1	0	0	1	1.81
17	0	0	0	0	1.71
18	0	−1	0	−1	1.09
19	−1	1	0	0	1.13
20	−1	0	0	−1	0.89
21	0	1	1	0	1.75
22	1	1	0	0	1.96
23	0	−1	0	1	1.36
24	1	0	1	0	1.66
25	0	1	−1	0	1.78
26	0	0	1	1	1.76
27	1	0	0	−1	1.47
28	0	0	−1	−1	1.58
29	−1	0	0	1	0.94

(3) $$\begin{array}{l} Y=1.65+0.30X_{1}+0.18X_{2}+0.014X_{3}+0.087X_{4}+0.19X_{1}X_{2}\\ \;-0.030X_{1}X_{3}+0.073X_{1}X_{4}+0.065X_{2}X_{3}-0.035X_{2}X_{4}\\ \;-0.037X_{3}X_{4}-0.31X_{1}^{2}-0.039X_{2}^{2}+0.015X_{3}^{2}-0.056X_{4}^{2} \end{array}$$

where *Y* is the yield rate of reducing sugar released from hydrolysis of rice straw by Tk-ChiA.

The suitability of the model was assessed by calculating several parameters (*p*, *F*-values, *R^2^*, lack of fit, and pure error) as shown in Supplementary Table S2. The *R^2^* of the model was 0.88, meaning that approximately 88% of the variability in the response was explained by the model. An *R^2^* between 0.75 and 1 indicates that the model has good accuracy (Moon et al., 2011). We used *p* to estimate the significance of the effect of each factor and the interactions between each independent variable. Based on the regression analysis (Supplementary Table S2), the linear coefficients, reaction time (*X*_1_) and temperature (*X*_2_) and the quadratic coefficients, $X_{1}^{2}$ was extremely significant (*p* < 0.001) and the interaction between time and temperature, *X*_1_*X*_2_, was significant (0.001 < *p* < 0.05). These results indicate that reaction time and temperature were the main factors affecting rice straw degradation by Tk-ChiA.

In addition, we also analyzed the effects of reaction time (*X*_1_), temperature (*X*_2_), pH (*X*_3_), and enzyme dosage (*X*_4_) on shrimp shell degradation by Tk-ChiA *via* orthogonal experimental designs (Supplementary Table S3). The range analysis indicated that reducing sugar production showed large variation under different levels *X*_1_. We further analyzed the *F*-values and *F* critical values to identify the main factors affecting shell degradation. The *F*-value of *X*_1_ (11.669) is larger than its *F* critical value (6.940), suggesting that the effect of *X*_1_ is significant (Supplementary Table S4). Therefore, reaction time was the most important factor affecting shrimp shell hydrolysis by Tk-ChiA.

### Optimal Level of Variables for Enhancing Rice Degradation

Reaction time (*X*_1_) and temperature (*X*_2_) are the main factors affecting rice straw degradation by Tk-ChiA. These two main effects (*X*_1_ and *X*_2_) were further optimized by central composite design (CCD) of response surface methodology (RSM). The levels for the variables (time and temperature) are listed in Table 3, and 13 runs designed *via* CCD were conducted (Table 4). The response optimized *via* CCD was estimated by the following second-order model:

**Table 3:** Range and levels of variables for CCD.

Factors	Levels				
	−1.414	−1	0	1	1.414
*X*_1_*–* time (h)	2.6	3	4	5	5.4
*X*_2_*–* temperature (°C)	54	60	70	80	84

**Table 4:** Experimental design and results of CCD.

Running number	*X*_1_	*X*_2_	Reducing sugar yield (%)
1	1	1	1.94
2	0	0	1.57
3	−1.414	0	1.32
4	−1	−1	0.88
5	0	0	1.57
6	−1	1	1.53
7	0	0	1.69
8	0	1.414	1.47
9	0	0	1.71
10	1	−1	1.45
11	0	−1.414	1.11
12	1.414	0	1.72
13	0	0	1.74

(4) $$Y=1.66+0.19X_{1}+0.21X_{2}-0.04X_{1}X_{2}-0.057X_{1}^{2}-0.17X_{2}^{2}$$

where *Y* is the yield rate of reducing sugar released from rice straw by Tk-ChiA hydrolysis. The results of the analysis of variance for the response surface quadratic model are listed in Supplementary Table S5. The *R^2^* of the model was 0.89, meaning that approximately 89% of the variability in the response was explained by the model. The *F*-value was 11.88, while the *p* was 0.0026, suggesting that the test model is a highly significant model of the reducing sugar yield rate. In addition, the effects of *X*_1_, *X*_2_, and $X_{2}^{2}$ were significant based on their *p* (Supplementary Table S5).

**Figure 5:**
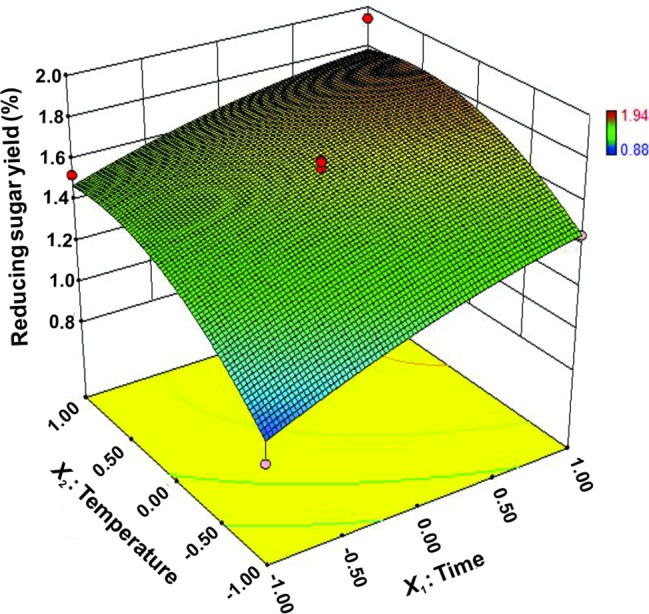
Response surface plot of reducing sugar released by rice straw as a function of time and temperature.

The interactions between *X*_1_ and *X*_2_ were also plotted on a response surface diagram (Figure 5), while other factors were maintained at a moderate (0) level to determine the optimum levels of *X*_1_ and *X*_2_ for the maximum response. As shown in Figure 5, reducing sugar production increased rapidly as the reaction time increased when the temperature was low, while it increased only slightly when the temperature was high. This effect corresponded to a loss of enzyme activity at high temperatures. However, the reducing sugar yield increased very rapidly when the temperature range was limited (<0.50 level). Reducing sugar production decreased at a high (>0.50) reaction temperatures (Figure 5), indicating that the enzyme lost activity at high temperatures. These findings suggest that the reducing sugar yield can be maximized by selecting an appropriate reaction time and temperature. The optimized values for the reaction time and temperature were determined by the model as follows: *X*_1_ (reaction time) = 5 h, *X*_2_ (temperature) = 65.4°C. The maximum predicted reducing sugar production (*Y*) was 1.68%.

## Discussion

Cellulases for degradation of cellulose have been reported in all three domains of life including eukaryote, prokaryote and archaea (Cabrera and Blamey, 2018). However, genes encoding cellulase are absent in *T. kodakarensis*, suggesting that *T. kodakarensis* cells need other sugars except cellulose degradation as carbon source to grow (Fukui et al., 2005). Based on the former researches, there are two hypothesized sugar degradation pathway of *T. kodakarensis*: (1) α-amylase (TK1884) (Tachibana et al., 1996) and two extracellular pullulanases (Tk0977 and Tk1774) (Han et al., 2013; Sun et al., 2015) from *T. kodakarensis* KOD1 were characterized. These enzymes can cleave α-1,4 and α-1,6 bonds of α-linked glucans and the hydrolytic products might be transported by an ABC type transport system and further enter into the glycolytic pathway. (2) The unique extracellular chitinase (Tk1765) (Tanaka et al., 1999, 2001; Aslam et al., 2017), *N*-acetylchitobiose deacetylase (TK1764) (Tanaka et al., 2004), exo-β-d-glucosaminidase (TK1754) (Tanaka et al., 2003), and the glucosamine-6-phosphate deaminase (TK1755) (Tanaka et al., 2005) were also characterized previously. Tk-CheA degraded chitin into the disaccharide *N*-,*N* = -diacetylchitobiose (GlcNAc)_2_ at outside of the cell, and (GlcNAc)_2_ is then hypothesized to be transported into cytoplasm of cell by an ABC type transport system. These enzymes form a novel chitinolytic pathway for chitin degradation and assimilation (Aslam et al., 2017). As cellulose is the most abundant polymer on earth, it is an excellent carbon source for microorganism growth. Actually, cellulases are frequently present in thermophilic archaea, such as *Pyrococcus* (Ando et al., 2002; Nakahira et al., 2013; Kataoka and Ishikawa, 2014; Kishishita et al., 2015) and *Thermoproteus* (Mardanov et al., 2011). Cellulases were also reported in the thermoacidophilic archaea *Acidilobus saccharovorans* (Mardanov et al., 2010), *Sulfolobus solfataricus* (Huang et al., 2005), and in the haloalkaliphilic archaeon *Haloarcula* (Shin et al., 2013; Li and Yu, 2013b). It is a keeping question why cellulase is absent in *T. kodakaraensis*.

The chitinase from the hyperthermophilic archaeon *T. kodakarensis* KOD1 (Tk-ChiA) is known to be capable of chitin hydrolysis (Tanaka et al., 1999, 2001; Aslam et al., 2017). Tk-ChiA possesses three binding domains that are considered to bind to chitin. The identical sequences of Tk-ChiA binding domains CBD2 and CBD3 are similar to the sequence of family II cellulose-binding domains (Tanaka et al., 1999) from various cellulases and chitinases (Tomme et al., 1998). CBD2 and CBD3 are known to be capable of binding chitin and cellulose (Hanazono et al., 2016). However, it is unknown whether Tk-ChiA catalyzes cellulose degradation. In this study, we expressed Tk-ChiA in *P. pastoris* and purified the enzyme. Purified Tk-ChiA was capable of degrading CMC-Na (Figures 1B, 2B), which corresponded to its ability to bind cellulose. However, it is unclear why the enzyme, Tk-ChiA, possesses the capacity to catalyze two different substrates.

Tk-ChiA is composed of dual catalytic domains on a single polypeptide. Both catalytic regions were classified in family 18 of glycosyl hydrolases *via* alignment of amino acid sequence (Tanaka et al., 1999). In *P. furiosus*, two chitinases (WP_011012376 and WP_011012377) were found and are homologous to the first and second catalytic domain of TK-ChiA, respectively (Oku and Ishikawa, 2006; Kreuzer et al., 2013). These two putative chitinase genes were expressed in *E. coli* and products were clearly identified to show chitinase activity (Gao et al., 2003; Oku and Ishikawa, 2006). In addition, the blast result of the sequence of TK-ChiA did not show similarity to catalytic domain of cellulase (data unshown). However, Tk-ChiA also showed cellulase activity in our studies. We hypothesized that Tk-ChiA evolved the secondary catalytic activity relying on its structure of chitin binding domains (CBD2 and CBD3). The secondary capacity of Tk-ChiA probably makes *T. kodakarensis* also utilizing cellulose as carbon source to compensate for the absence of cellulase in the species. The biochemical properties of the individual catalytic domains (Tk-ChiA1 and Tk-ChiA2) hydrolyzing chitin were studied in detail and showed that the amino-terminal domain catalyzes an exo-type cleavage of the chitin chain, while the carboxy-terminal domain catalyzes an endo-type cleavage of the chitin chain (Tanaka et al., 2001). The activity of Tk-ChiA1 and Tk-ChiA2 without binding domain decreased apparently, indicating the important role of binding domains. We will study the cellulose degradation activity of each catalytic and binding domain combinations to investigate how Tk-ChiA catalyzes cellulose in the future.

The purified Tk-ChiA showed cellulose hydrolysis activity within a wide range of high temperatures (50–80°C), and its optimal cellulose catalytic temperature was 65°C (Figure 3A), which was lower than its optimal chitin catalytic temperature (80°C). Tk-ChiA showed nearly 100% of its maximum cellulose hydrolysis activity after pre-incubation at 70°C for 4 h, and it showed more than 40% of its maximum activity after pre-incubation at 100°C for 4 h (Figure 3C). The effect of high temperatures on cellulose hydrolysis by Tk-ChiA is similar to the effect of high temperatures on chitin degradation by Tk-ChiA (Tanaka et al., 2001). Industrial enzymatic processes such as saccharification are generally performed at high temperatures for high efficiency (Nigam, 2013). The thermostability and dual catalytic ability of Tk-ChiA provide advantages in industrial applications such as shrimp shell and rice straw degradation. The *P. pastoris* system was selected to express Tk-ChiA due to its advantages over other systems. The only major protein band detected in the supernatant of the fermentation broth of the yeast carrying the Tk-ChiA expression vector was Tk-ChiA (Figure 1). In addition, the specific activity of crude enzyme and purified enzyme are similar, suggesting that this enzyme did not need further purification. Enzyme purification is expensive, so this finding also indicates an advantage of Tk-ChiA in industrial applications.

Shell and straw are abundant wastes. For example, shrimp shells are discarded as rubbish and large amounts of rice straw are left on the field and burned off after harvest, causing environmental problems. However, shrimp shell and rice straw contain abundant chitin (Zhang et al., 2012) and cellulose (Fan et al., 2013), respectively, which can serve as low-cost feedstocks for the production of fuel ethanol (Biswas et al., 2006). Therefore, nonuse of these wastes results in a loss of a potentially valuable source of energy (Mishra and Sen, 2011). With recent developments in fermentation technology, environmentally friendly processes such as enzymatic chitin and cellulose degradation have attracted significant interest. Shrimp shell and rice straw were obviously degraded by Tk-ChiA according to our SEM and FT-IR analyses (Figure 4). Reaction time and temperature were determined to be the major factors affecting degradation of shrimp shell and rice straw by Tk-ChiA to release reducing sugar (Table 2; Supplementary Tables S2–S4). Therefore, these factors were optimized by CCD of RSM to maximize the rate of reducing sugar production by Tk-ChiA (Table 4, Figure 5). Using the optimized reaction parameters reported here, Tk-ChiA could be utilized for cost-efficient rice straw degradation in industrial applications.

In conclusion, the thermostable enzyme Tk-ChiA has dual catalytic functions in cellulose and chitin degradation and is thus suitable for shell and straw degradation in industrial applications. CCD of RSM is a valuable strategy that can be utilized to enhance rice straw degradation to release reducing sugar.

## Data Availability

No datasets were generated or analyzed for this study.

## Author Contributions

ZL and S-HZ designed the research. LC and ZL performed research and interpreted data. MS and YW supported the experiments. ZL wrote the paper.

### Conflict of Interest Statement

The authors declare that the research was conducted in the absence of any commercial or financial relationships that could be construed as a potential conflict of interest.

## Footnotes

^1^http://www.cazy.org/

^2^https://tools.thermofisher.com/content/sfs/manuals/pich_man.pdf

^3^https://tools.thermofisher.com/content/sfs/manuals/pich_man.pdf
