# Supplementary material for: An Archaeal Chitinase With a Secondary Capacity for Catalyzing Cellulose and Its Biotechnological Applications in Shell and Straw Degradation
Source: Front Microbiol. 2019 Jun 11;10:1253. doi: 10.3389/fmicb.2019.01253 (PMC6579819; doi:10.3389/fmicb.2019.01253)
Supplement: Supplementary file 1 [file Table_1.DOCX]

Supplementary Material

**Supplementary figures**

**Fig. S1** Sequence analysis of Tk-ChiA (Tk1765). In the beginning of 28 amino acids were predicted to be signal peptide highlighted in yellow. Tk-ChiA possesses 3 substrate binding domains (CBD1, CBD2 and CBD3) that were boxed. CBD1 (chitin-binding domain) was highlighted in green and CBD2/3 (chitin/cellulose-binding domains) were highlighted in purple. The enzyme catalytic domains (GH18_ChiA1, 2) were shown by underline and dotted line, respectively.

**Fig. S1**

**
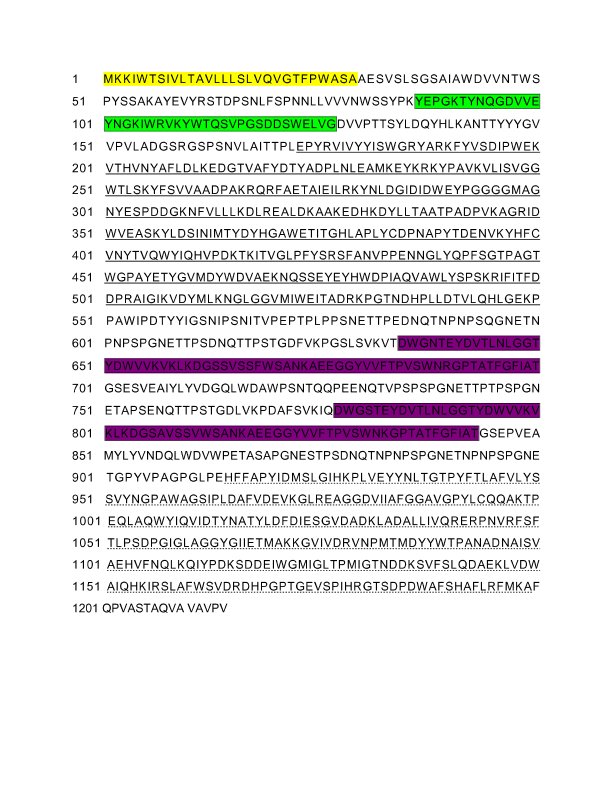
**

**Supplementary tables**

**Table S1** Different pHs buffers used in this study

| Buffers | Concentration (mM) | pH |
| --- | --- | --- |
| citrate–phosphate buffer | 50 | 3.0-5.0 |
| MES-NaOH buffer | 50 | 6.0-7.0 |
| Tris-HCl buffer | 50 | 8.0 |
| glycine-NaOH buffer | 50 | 9.0-11.0 |

**Table S2** Variance analysis of statistical experiments designs for rice straw degradation

| Source | Sum of  Squares | df | Mean  Square | F | *p-value* | Saliency analysis |
| --- | --- | --- | --- | --- | --- | --- |
| Model | 2.43 | 14 | 0.17 | 7.34 | 0.0003 | ** |
| *X*_1_ | 1.09 | 1 | 1.09 | 45.9 | < 0.0001 | ** |
| *X*_2_ | 0.39 | 1 | 0.39 | 16.43 | 0.0012 | ** |
| *X*_3_ | 2.41E-03 | 1 | 2.41E-03 | 0.1 | 0.7544 |  |
| *X*_4_ | 0.09 | 1 | 0.09 | 3.81 | 0.0713 |  |
| *X*_1_*X*_2_ | 0.15 | 1 | 0.15 | 6.43 | 0.0238 | * |
| *X*_1_*X*_3_ | 3.60E-03 | 1 | 3.60E-03 | 0.15 | 0.7024 |  |
| *X*_1_*X*_4_ | 0.021 | 1 | 0.021 | 0.89 | 0.3619 |  |
| *X*_2_*X*_3_ | 0.017 | 1 | 0.017 | 0.71 | 0.4123 |  |
| *X*_2_*X*_4_ | 4.90E-03 | 1 | 4.90E-03 | 0.21 | 0.656 |  |
| *X*_3_*X*_4_ | 5.63E-03 | 1 | 5.63E-03 | 0.24 | 0.6334 |  |
| *X*_1_^2^ | 0.62 | 1 | 0.62 | 26.33 | 0.0002 | ** |
| *X*_2_^2^ | 9.70E-03 | 1 | 9.70E-03 | 0.41 | 0.5324 |  |
| *X*_3_^2^ | 1.48E-03 | 1 | 1.48E-03 | 0.062 | 0.8064 |  |
| *X*_4_^2^ | 0.02 | 1 | 0.02 | 0.86 | 0.3682 |  |
| Residual | 0.33 | 14 | 0.024 |  |  |  |
| Lack of Fit | 0.27 | 10 | 0.027 | 1.67 | 0.3288 | not significant |
| Pure Error | 0.064 | 4 | 0.016 |  |  |  |
| Cor Total | 2.76 | 28 |  |  |  |  |

Note：** (highly significant)，*P*＜0.01；* (significant)，0.01＜*P*＜0.05.

**Table S3** Orthogonal experimental designs and results of shell degradation

| Running  number |  | Factors | |  | Reducing sugar yield (%) | | |
| --- | --- | --- | --- | --- | --- | --- | --- |
|  | *X*_1_ | *X*_2_ | *X*_3_ | *X*_4_ |  |  |  |
| 1 | -1 | -1 | -1 | -1 | | 0.57 |  |
| 2 | -1 | 0 | 0 | 0 | | 0.79 |  |
| 3 | -1 | 1 | 1 | 1 | | 1.18 |  |
| 4 | 0 | -1 | 0 | 1 | | 1.37 |  |
| 5 | 0 | 0 | 1 | -1 | | 1.54 |  |
| 6 | 0 | 1 | -1 | 0 | | 1.81 |  |
| 7 | 1 | -1 | 1 | 0 | | 1.97 |  |
| 8 | 1 | 0 | -1 | 1 | | 3.12 |  |
| 9 | 1 | 1 | 0 | -1 | | 2.31 |  |
| Mean value1 | 0.847 | 1.303 | 1.833 | 1.473 | |  |  |
| Mean value2 | 1.573 | 1.817 | 1.490 | 1.523 | |  |  |
| Mean value3 | 2.467 | 1.767 | 1.563 | 1.890 | |  |  |
| Range | 1.620 | 0.514 | 0.343 | 0.417 | |  |  |

**Table S4** Variance analysis of shell degradation orthogonal experiment

| Factors | *X*_1_ | *X*_2_ | *X*_3_ | *X*_4_ |
| --- | --- | --- | --- | --- |
| Sum of Squares | 3.95 | 0.481 | 0.196 | 0.311 |
| df | 2 | 2 | 2 | 2 |
| F | 11.669 | 1.421 | 0.579 | 0.919 |
| F critical value | 6.940 | 6.940 | 6.940 | 6.940 |
| Saliency analysis | * |  |  |  |

**Table S5** Variance analysis of CCD

| Source | Sum of  Squares | df | Mean  Square | F | *p-value* | Saliency analysis |
| --- | --- | --- | --- | --- | --- | --- |
| Model | 0.86 | 5 | 0.17 | 11.88 | 0.0026 | ** |
| *X*_1_ | 0.30 | 1 | 0.30 | 20.66 | 0.0027 | ** |
| *X*_2_ | 0.34 | 1 | 0.34 | 23.52 | 0.0019 | ** |
| *X*_1_*X*_2_ | 6.400E-003 | 1 | 6.400E-003 | 0.44 | 0.5271 | * |
| *X*_1_^2^ | 0.022 | 1 | 0.022 | 1.55 | 0.2532 | * |
| *X*_2_^2^ | 0.21 | 1 | 0.21 | 14.20 | 0.0070 | ** |
| Residual | 0.10 | 7 | 0.014 |  |  |  |
| Lack of Fit | 0.075 | 3 | 0.025 | 3.87 | 0.1120 | not significant |
| Pure Error | 0.026 | 4 | 6.480E-003 |  |  |  |
| Cor Total | 0.96 | 12 |  |  |  |  |

Note：** (highly significant)，*P*＜0.01；* (significant)，0.01＜*P*＜0.05.

**Supplementary Table S6:** Yield of Tk-ChiA production

| Purification steps | Volume (mL) | Total protein (mg) | Total activity (U) | Specific activity (U/mg) |  |
| --- | --- | --- | --- | --- | --- |
| Crude enzyme | 50 | 15.3 | 2524.4 | 164.993 |  |
| Purified enzyme | 10 | 13.05 | 2461.2 | 188.598 |  |
